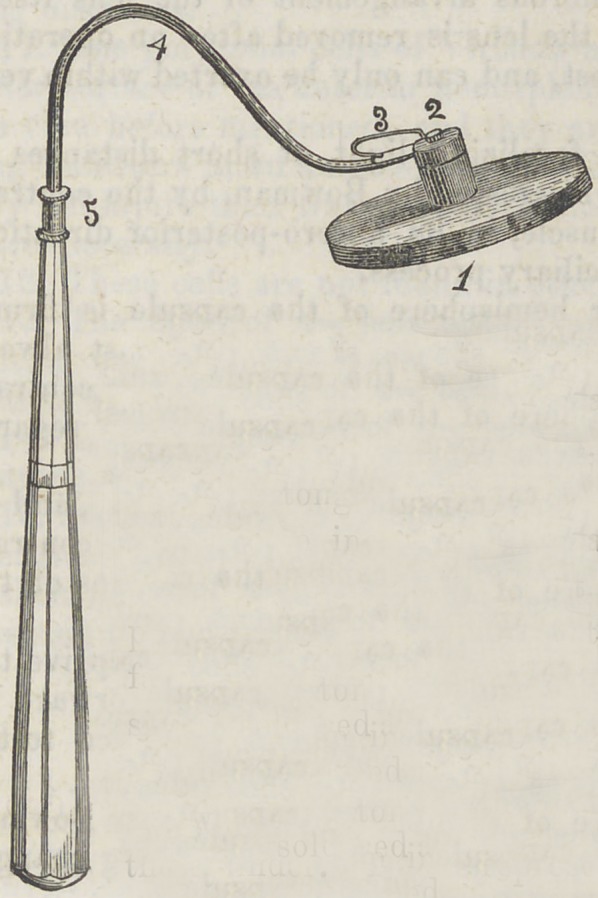# Merriam’s Tongue Holder

**Published:** 1860-01

**Authors:** 


### MERRIAM'S TONGUE HOLDER.

The accompanying engraving represents an instrument invented and made by Dr. A. Merriam, for holding the tongue down during the operation of filling. Its construction is well exhibited by the cut, and scarcely requires a description. Fig. 1 represents the plate of the instrument that rests upon the tongue, elliptical in form, its diameter an inch by an inch and a fourth, respectively. It is made of silver plate, about No. 22; it presents a plain surface to the tongue, though a concave surface might be better in some cases. Upon the upper side is soldered a cylindrical peice of silver, to which which is attached the shaft of the instrument, by a screw passing through the head of the shaft into the cylinder. Fig. 2 represents the attachment of the shaft. No. 3 is a spring, acting upon the joint, giving better control of the instrument; by this arrangement, the shaft of the instrument can be moved to either side of the mouth without changing the position of the plate that rests upon the tongue. Fig. 4 is the shaft of the instrument.

## Figures and Tables

**Figure f1:**